# Intravenous thrombolysis versus antiplatelet standard care for patients with mild acute ischemic stroke: a systematic review and meta-analysis

**DOI:** 10.3389/fmed.2026.1780490

**Published:** 2026-03-11

**Authors:** Yitao Zhou, Yangbin Zhou, Ganying Hunag, Hongmei Wang

**Affiliations:** 1 School of Nursing, Zhejiang Chinese Medical University, Hangzhou, Zhejiang, China; 2 Department of Emergency, Affiliated Hangzhou First People’s Hospital, School of Medicine, Westlake University, Hangzhou, Zhejiang, China

**Keywords:** antiplatelet standard care, intravenous thrombolysis, meta-analysis, mild acute ischemic stroke, review

## Abstract

**Objectives:**

To evaluate the safety and efficacy of intravenous thrombolysis (IVT) versus standard antiplatelet therapy in patients with minor acute ischemic stroke (AIS).

**Methods:**

A systematic review and meta-analysis of databases, including PubMed, Embase, Web of Science, and Cochrane Library, up to 27 June 2025. Inclusion criteria were randomized clinical trials comparing IVT with standard antiplatelet care in patients with mild acute ischemic stroke. Exclusions included non-randomized studies, observational studies, non-interventional trials, meeting abstracts, duplicates, studies with overlapping data, and non-English language studies. This study followed the Preferred Reporting Items for Systematic Reviews and Meta-Analyses (PRISMA) guidelines. Fixed-effects or random-effects model meta-analysis were used to analyze the pooled data. Main outcomes and measures: Rates of functional independence (modified Rankin Scale score ≤ 1 or ≤ 2), 90-day mortality, sICH.

**Results:**

Four randomized clinical trials, involving 3,405 initially enrolled patients, were included in the analysis. A lower rate of functional independence was observed in the IVT group (mRS ≤ 1, relative risk [RR], 0.97 [95% CI, 0.94–1.00]; mRS ≤ 2, RR, 0.97 [95% CI, 0.95–0.99]). Higher 90-day mortality rates (RR, 2.42 [95% CI, 1.39–4.20]) and sICH rates (RR, 4.90 [95% CI, 1.67–14.40]) were observed in the IVT group. All outcomes reported in this analysis had low heterogeneity.

**Conclusion and relevance:**

Our findings suggest that intravenous thrombolysis for mild acute ischemic stroke yields no benefit and may pose additional risks compared to antiplatelets standard care. More large clinical randomized controlled trials are still needed in the future to validate our results.

**Systematic review registration:**

[https://www.crd.york.ac.uk/prospero/], identifier [CRD42025643646].

## Introduction

Intravenous thrombolysis, as an important treatment for acute ischemic stroke, has been validated in multiple studies for its efficacy and safety (1–3). About half of ischemic stroke patients present with mild neurological deficits, defined as a National Institutes of Health Stroke Scale (NIHSS) score of five or less (4). A meta-analysis of individual patient data from randomized trials indicated that alteplase significantly increases the odds of favorable outcomes in ischemic stroke patients, regardless of stroke severity, particularly when administered intravenously within 4.5 h of symptom onset (5). Currently, based on published studies, all international guidelines consistently recognize that intravenous administration of alteplase within 4.5 h of symptom onset is a safe and effective treatment for patients with AIS (6–8). It is noteworthy that some studies have emphasized this recommendation is conditional—intravenous administration of alteplase is advised only for patients with minor stroke presenting with disabling symptoms who can be treated within 4.5 h of symptom onset. Conversely, intravenous thrombolysis is not recommended for patients with mild, non-disabling stroke (9, 10). While current guidelines present ambiguous recommendations regarding intravenous thrombolysis for minor acute ischemic stroke, they also acknowledge the potential benefits of the treatment (7). However, intravenous thrombolysis didn’t offer an advantage over antiplatelet therapy for patients with mild acute ischemic stroke. The PRISMS trial, which enrolled patients with minor ischemic stroke in 75 stroke hospitals in the United States and compared the efficacy of alteplase with aspirin, showed that alteplase did not increase the likelihood of a good functional outcome (11). Similarly, the ARAMIS trial showed that intravenous thrombolytic therapy (alteplase) was not superior to antiplatelet therapy (12). More importantly, the results of the TEMPO-2 study, published in 2024, showed no benefit and some degree of harm from intravenous thrombolytic therapy with Tenecteplase (13). A recently published PUMICE trial also found that urokinase thrombolysis was not superior to standard antiplatelet therapy, but had a similar safety profile (14).

According to a 2024 meta-analysis (34), in patients with mild AIS, early use of antiplatelet therapy was significantly associated with a reduced risk of symptomatic intracranial hemorrhage (sICH) compared with IVT, but was not significantly associated with other outcomes. However, with the recent publication of high-quality clinical randomized controlled trials and the inclusion of a large number of observational studies in previous studies, which may have introduced risk of bias, it is necessary to update the meta-analysis on this topic. Most current guidelines still recommend the administration of intravenous thrombolysis as early as possible for ischemic stroke, regardless of the severity of the patient’s condition (15–17). In light of the existing conflicting evidence and the necessity of updating, therefore, we performed a systematic review and meta-analysis of RCTs comparing intravenous thrombolysis and antiplatelet standard care.

## Methods

### Search strategy

The present meta-analysis was conducted the Preferred Reporting Items for Systematic Reviews and Meta-Analyses (PRISMA) guidelines (18). PROSPERO Registration Number: CRD42025643646. We extensively searched multiple databases including Cochrane Library, Embase, PubMed, and Web of Science to identify relevant studies published before 27 June 2025. The PubMed search strategy included the terms “ischemic stroke,” “mild OR minor,” “intravenous thrombolysis,” “antiplatelet.” Additionally, we manually searched the references of selected studies and current field reviews to identify relevant literature. After Endnote software eliminated duplicate studies, the remaining articles were carefully reviewed, the complete search strategy in Supplementary Table 1.

### Screening process and eligibility criteria

Two researchers (Y. Zhou and H. Wang) independently performed the initial screening of titles and abstracts using a blinded approach, following established criteria. Studies that met the initial criteria were then subjected to a detailed full-text review. During both screening phases, the senior researcher (H. Wang) played a key role in resolving any disagreements. We included all randomized controlled trials (RCTs) that matched our predefined PICO framework (patient or problem; intervention or exposure; comparison or control; and outcome). The focus was on patients with mild acute ischemic stroke receiving intravenous thrombolysis and antiplatelet therapy, mild AIS was defined as National Institutes of Health Stroke Scale (NIHSS) scores ≤ 5 (19, 20). The intervention method was intravenous thrombolysis, compared against standard antiplatelet treatment. Main outcomes included functional independence, defined as a modified Rankin Scale (mRS) score (scores range from 0 to 6, with higher scores indicating greater disability) (21, 22). For a more precise analysis, mRS ≤ 1 represents symptomatic but non-disabled and mRS ≤ 2 represents disabled but independent, both represent good functional independence (22). Secondary outcomes included 90-day mortality, and symptomatic intracranial hemorrhage (sICH). Exclusions comprised non-randomized studies, observational research, non-interventional trials, meeting abstracts, duplicate publications, studies with overlapping data, and non-English language studies.

### Data extraction

After completing the literature screening, only four studies met the inclusion criteria. Two researchers (Yb. Zhou and Y. Zhou) were assigned to extract data using Excel, focusing on key details such as study characteristics, baseline patient information, and the predefined outcomes. To ensure accuracy, a third researcher (G. Huang) reviewed the extracted data, addressing any inconsistencies and facilitating consensus among the team. To identify potential differences in baseline features, we analyzed multiple aspects of each study, including study design, group allocation, age and sex distribution, medical history, racial and ethnic composition, baseline National Institutes of Health Stroke Scale (NIHSS) scores, Alberta Stroke Program Early CT (ASPECT) scores, time from symptom onset to treatment initiation, blood glucose levels, and the Trial of Org 10172 in Acute Stroke Treatment (TOAST) classification.

### Risk of bias assessment

Two reviewers (Y. Zhou and G. Huang) evaluated the risk of bias in the included RCTs using the Cochrane Risk of Bias Tool. This tool examines potential biases across several domains (23): Random sequence generation (selection bias); Allocation concealment (selection bias); Blinding of participants and personnel (performance bias); Blinding of outcome assessment (detection bias); Incomplete outcome data (attrition bias); Selective reporting (reporting bias); Other bias. Studies with a high risk of bias would be excluded from the analysis; however, no such studies were identified in the present review.

### Statistical analysis

In this study, we conducted a meta-analysis using Stata software, version 15 (64-bit), along with the meta statistical package. Risk ratios (RRs) with 95% confidence intervals (CIs) were calculated for binary outcomes. Heterogeneity was evaluated using the I^2^ statistic, I^2^ values exceeding 50% indicating high heterogeneity. An I^2^ < 50% indicated low heterogeneity and a fixed-effect model was employed. Furthermore, I^2^ > 50% indicated high heterogeneity, and thus a random-effect model was utilized. To examine the impact of individual studies on the overall results, we performed influence analysis using the leave-one-out approach. However, due to the small number of studies included in each analysis (fewer than seven), we were unable to assess publication bias using Egger’s regression test or perform meta-regression (23).

## Results

### Study selection and evaluation

**FIGURE 1:**
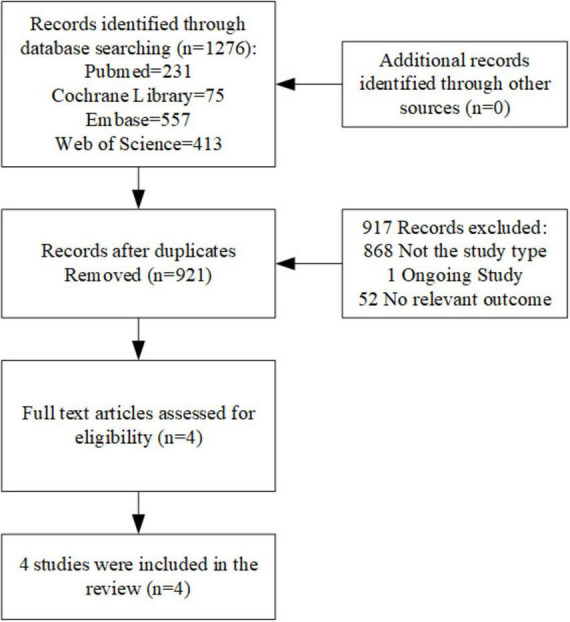
Flow diagram: the study selection procedure.

The initial search across databases identified 1,276 records, comprising 231 from PubMed, 75 from the Cochrane Library, 557 from Embase, and 413 from Web of Science. After removing duplicates and screening titles and abstracts, 921 studies remained for further evaluation. Of these, 917 were excluded during the title and abstract screening phase, leaving four studies for full-text review. All four studies met the inclusion criteria and were included in the quantitative synthesis (Figure 1). These studies, published between 2018 and 2025, were conducted in multiple countries, including the US, China, Australia, Austria, Brazil, Canada, Finland, Ireland, New Zealand, Singapore, Spain, and the UK. The study included a total of 3,405 patients enrolled in the primary analysis. The key characteristics of the included studies are summarized in Table 1.

**TABLE 1:** Baseline characteristics of included studies.

Study		Khatri et al. (11)		Chen et al. (12)		Coutts et al. (13)		Xiong et al. (14)	
Design		RCT		RCT		RCT		RCT	
Groups		Alteplase group	Antiplatelet therapy group (aspirin)	Alteplase group	Dual antiplatelet therapy group (clopidogrel and aspirin)	Tenecteplase group	Antiplatelet therapy group (depend on the local investigator)	Prourokinase group	Antiplatelet therapy group (depend on the local investigator).
Male/female		77/79	92/65	240/110	256/113	244/188	272/180	479/244	469/254
Age	Mean	62	61	63.6	64.3	71.3	70.6	65.5	65.3
	SD	14	13	11.2	10.4	13.4	13.4	11.6	10.5
Medical history	Hypertension	126	124	169	211	265	261	537	524
	Hyperlipidemia	114	114	N/A	N/A	180	172	207	215
	Diabetes mellitus	57	44	86	101	82	86	171	192
	Previous stroke	28	24	77	82	72	85	191	200
	Atrial fibrillation	23	17	N/A	N/A	91	78	40	35
	Congestive heart failure	N/A	N/A	N/A	N/A	16	18	N/A	N/A
	Chronic renal failure	N/A	N/A	N/A	N/A	22	17	N/A	N/A
	Peripheral vascular disease	N/A	N/A	N/A	N/A	13	15	N/A	N/A
	Coronary heart disease	N/A	N/A	N/A	N/A	N/A	N/A	150	157
Race and ethnicity	White	117	126	N/A	N/A	371	382	N/A	N/A
	Black	35	27	N/A	N/A	6	7	N/A	N/A
	American Indian or Alaska Native	1	3	N/A	N/A	N/A	N/A	N/A	N/A
	Asian	0	1	350	369	40	42	723	723
	≥2 races	1	0	N/A	N/A	N/A	N/A	N/A	N/A
	Pacific Islander	N/A	N/A	N/A	N/A	1	0	N/A	N/A
	Unknown	2	0	N/A	N/A	10	16	N/A	N/A
	Hispanic or Latino ethnicity	14	18	N/A	N/A	N/A	N/A	N/A	N/A
Baseline NIHSS score	Mean	2.3	2	2	2	2	2	3	2
	SD	1.2	1.2	1.5	1.5	1.5	1.5	1.5	1.5
ASPECTS score	Mean	9.9	9.9	N/A	N/A	10	10	10	10
	SD	0.6	0.6	N/A	N/A	0.2	0.2	0.2	0.2
Time from onset of symptoms to receipt of assigned treatment (min)	Mean	155.7	151.8	177.2	182	296.2	295.4	184.5	181.5
	SD	31.4	35.9	73	71.4	207.5	212.7	61.7	63.1
Glucose level (mmol/L)	Mean	7.8	7.3	6.6	6.7	6.4	6.4	N/A	N/A
	SD	4.1	3.4	2	2.2	0.7	0.7	N/A	N/A
TOAST classification	Large artery atherosclerosis	N/A	N/A	46	54	N/A	N/A	290	281
	Cardioembolic	N/A	N/A	1	1	N/A	N/A	14	22
	Small artery occlusion	N/A	N/A	79	87	N/A	N/A	389	390
	Undetermined cause	N/A	N/A	221	225	N/A	N/A	17	19
	Other determined cause	N/A	N/A	3	2	N/A	N/A	9	11

### Risk of bias

The four studies included in our analysis, along with their bias assessments across different domains using the Cochrane Risk of Bias Tool, are presented in Supplementary Figure 1. Our evaluation indicated that all studies carried a moderate risk of bias, with particular concerns identified in the domains of attrition bias and reporting bias.

### Functional independence

**FIGURE 2:**
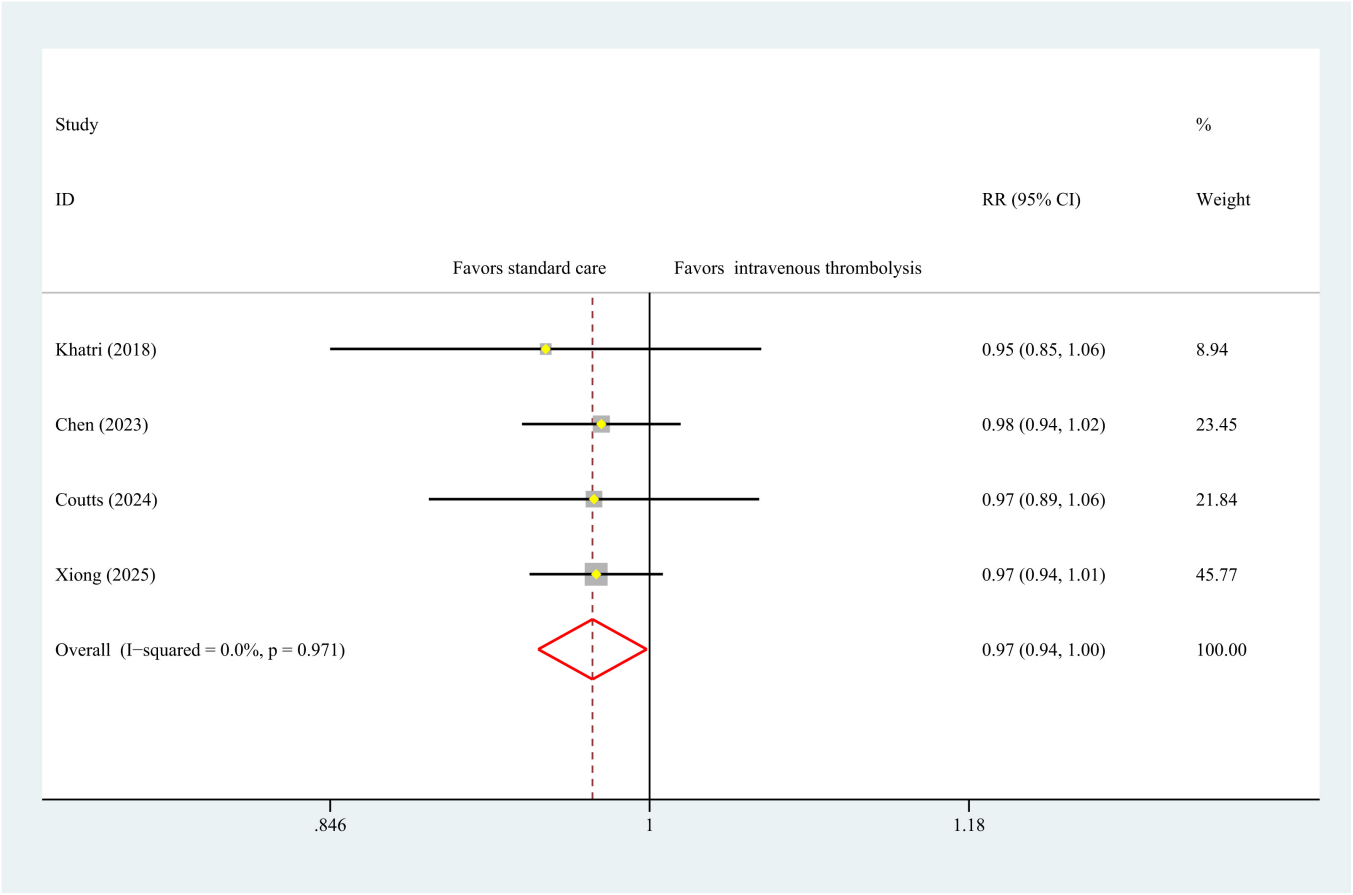
Forest plot. Meta-analysis of the incidence of 90-day mRS score = 0–1 score. mRS, modified Rankin Scale.

**FIGURE 3:**
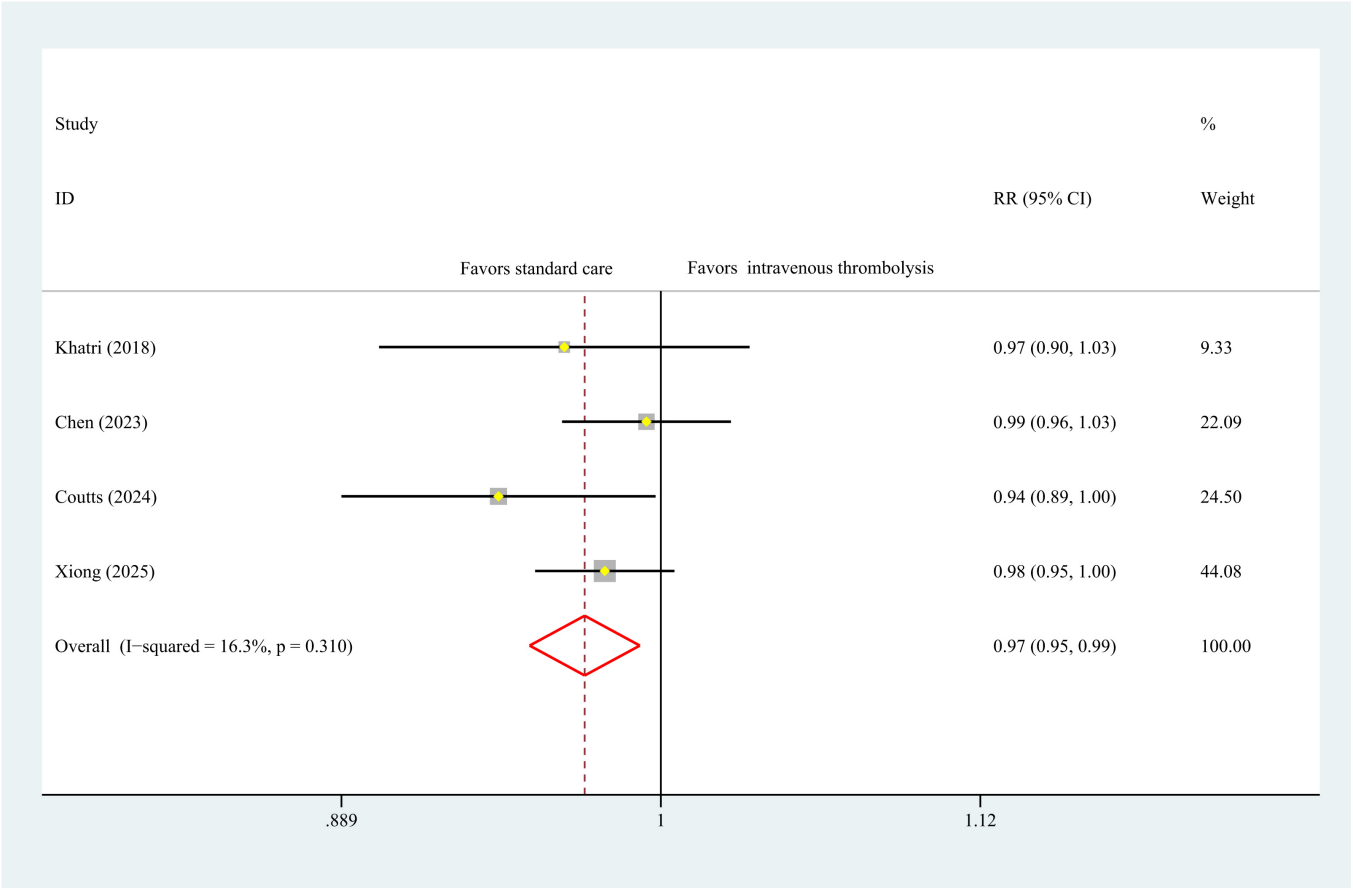
Forest plot. Meta-analysis of the incidence of 90-day mRS = 0–2 score.

Four studies with 3,362 patients provided data on 90 days functional independence (mRS score = 0–1), a meta-analysis using a fixed-effects model indicated statistically significant differences in intravenous thrombolysis and antiplatelet therapy (RR, 0.97 [95% CI, 0.94–1.00]; *P* = 0.04), with no heterogeneity (I^2^ = 0%, *P* = 0.97), Figure 2. As for the mRS score = 0–2, we observed the same statistically differences tendency (RR, 0.97 [95% CI, 0.95–0.99]; *P* = 0.01), with low heterogeneity (I^2^ = 0%, *P* = 0.97), Figure 3.

### 90 -day mortality

**FIGURE 4:**
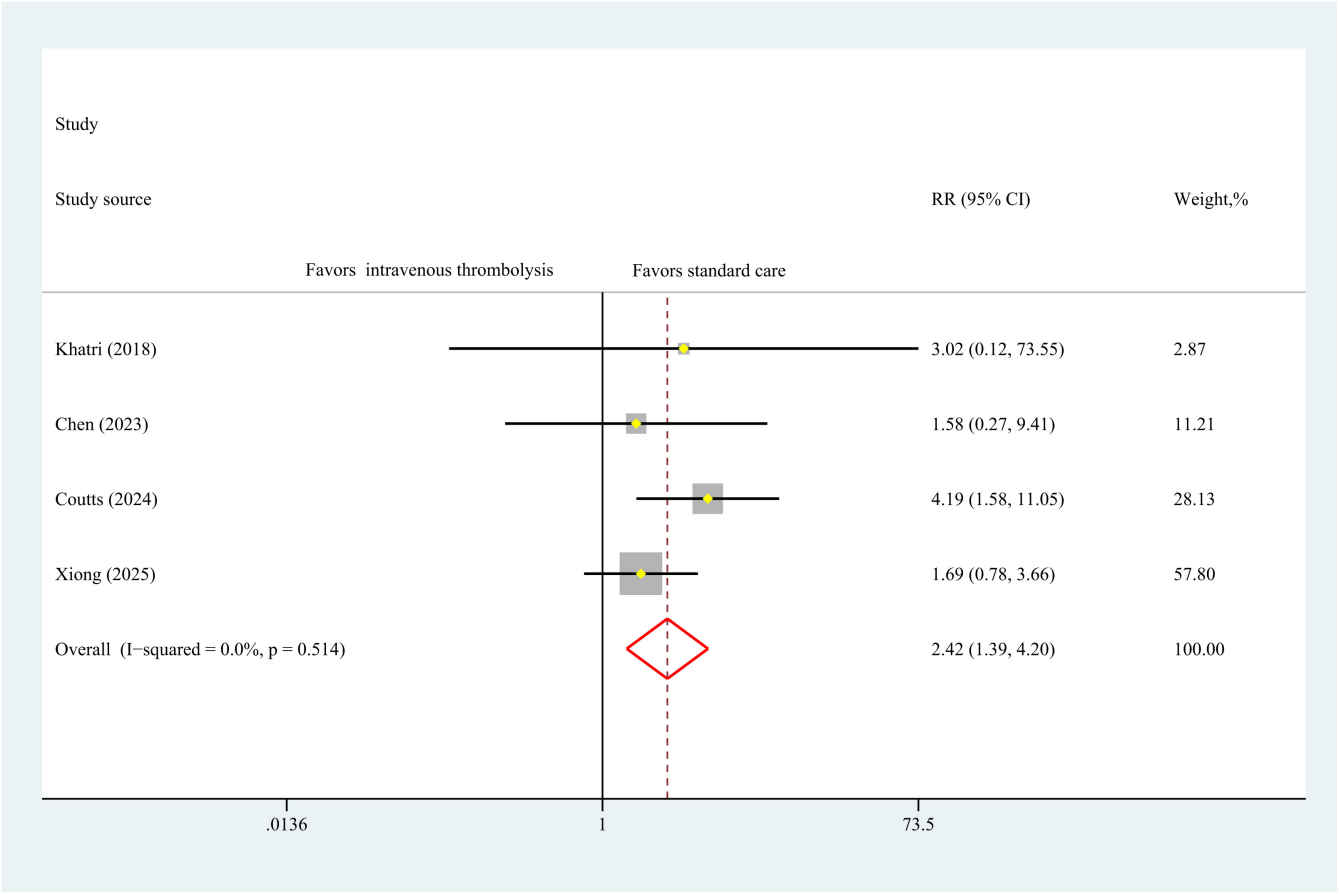
Forest plot. Meta-analysis of the incidence of 90-day mortality.

The results of 90-day mortality data were obtained from four studies encompassing 3,362 patients. As seen in Figure 3, IVT was associated with significant higher 90-day mortality compared to antiplatelet therapy (RR, 2.42 [95% CI, 1.39–4.20]; *P* < 0.01), with no heterogeneity (I^2^ = 0%, *P* = 0.51) in a fixed-effect model analysis (Figure 4).

### Symptomatic intracranial hemorrhage

**FIGURE 5:**
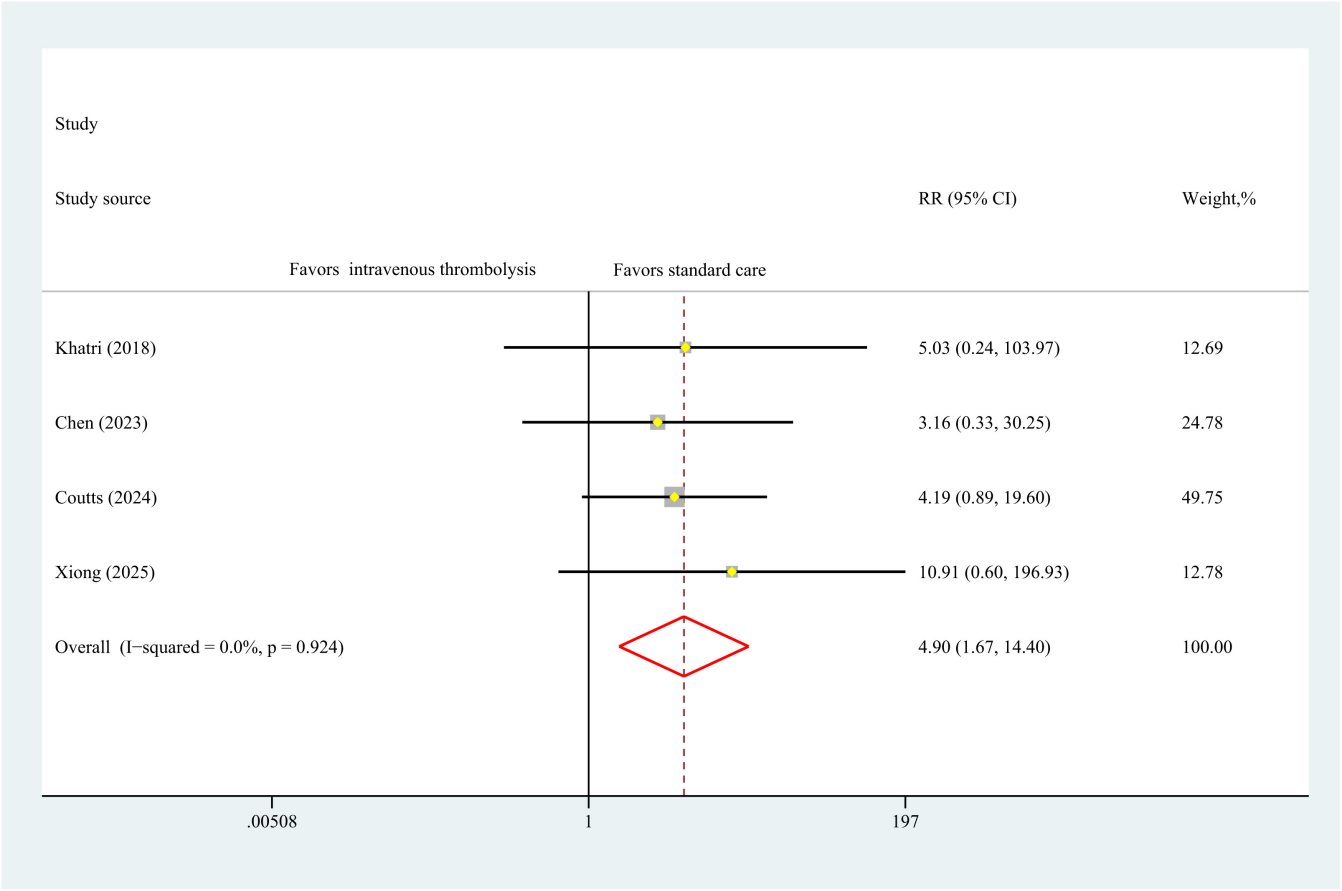
Forest plot. Meta-analysis of the incidence of sICH. sICH, symptomatic intracranial hemorrhage.

The results of sICH data were obtained from 4 studies encompassing 3,362 patients. As seen in Figure 5, statistically significant difference in sICH was observed across intravenous thrombolysis and antiplatelet therapy (RR, 4.90 [95% CI, 1.67–14.40]; *P* < 0.01), with no heterogeneity (I^2^ = 0%, *P* = 0.92) in a fixed-effect model analysis.

### Leave-one-out influence analysis

For the functional independence outcome (mRS ≤ 1), removing the study by Coutts et al. brought the results to be more significant (RR, 0.97 [95% CI, 0.94–0.99]), in Supplementary Figure 2; nevertheless, as for the functional independence outcome (mRS ≤ 2), excluding any of the other studies would not result in eliminating significance except the study by Coutts et al. (RR, 0.98 [95% CI, 0.96–1.00]), in Supplementary Figure 3. As for the 90-day mortality outcome, removing the study by Coutts et al resulted in eliminating significance (RR, 1.72 [95% CI, 0.86–3.44]), in Supplementary Figure 4. For the sICH outcomes, removing any study did not have a tangible effect on significance (Supplementary Figure 5).

## Discussion

Our meta-analysis compared the safety and efficacy of intravenous thrombolytic therapy with standard antiplatelet therapy for mild acute ischemic stroke. At the same time, the findings suggested that intravenous thrombolytic therapy provides no benefit and was associated with a lower probability of functional independence and increases the probability of 90-day mortality and sICH. These findings supported the use of standard antiplatelet therapy rather than intravenous thrombolysis for minor acute ischemic stroke. Obviously, this was inconsistent with the results of previous studies (10, 20, 24).

On the one hand, previous studies have shown that IV thrombolysis could benefit patients. Moawad et al. pooled and analyzed 93,057 patients with mild acute ischemic stroke and demonstrated that IVT improved functional independence with NIHSS scores without increasing the risk of sICH or death (20). However, this study included a large number of observational studies and did not use antiplatelet therapy as a control group. On the other hand, clinical randomized controlled trials have shown that IV thrombolysis did not benefit compared to antiplatelet therapy, and to some extent brought disadvantages. PRISMS was the first RCT to assess the effect of an intravenous thrombolytic drug (alteplase) on minor ischemic stroke (11). The study found that alteplase did not improve the 90-day probability of functional independence compared with aspirin. During the same time period, the MINOR trial recruited mild AIS patients in Italy (25). Unfortunately, this trial was stopped in 2017 because it did not meet its recruitment goals. In fact, it recruited only 30 of the expected 300 patients. This was most likely because many neurologists in Italy with guidelines were more accustomed to use alteplase for minor strokes in their daily practice (25). Analysis of these randomly assigned 30 patients revealed no significant differences between the two groups. However, the sample size was too small for a valid comparison. Since then, the American Heart Association (AHA) and American Stroke Association (ASA) guidelines had also recommended that intravenous thrombolysis could be considered in patients with minor strokes within 3 h of symptom onset, which was the choice of most neurologists (8). Since then, successive ARAMIS trial, TEMPO-2 trial, and PUMICE trial have been conducted in validation to explore the same issues (12–14).

It is noteworthy that the four included RCTs employed three pharmacologically distinct thrombolytic agents. The PRISMS and ARAMIS used alteplase (0.9 mg/kg, with 10% as a bolus and 90% infused over 1 h), TEMPO-2 used tenecteplase (0.25 mg/kg single bolus), and PUMICE used prourokinase (35 mg, 15 bolus + 20-mg infusion over 30 min) (11–14). Tenecteplase has a longer half-life, greater fibrin specificity, and less systemic fibrinogen depletion compared with alteplase (26), whereas prourokinase is a specific plasminogen activator with a potentially lower bleeding risk (27). Similarly, the antiplatelet regimens in the control arms varied across trials. The PRISMS used aspirin monotherapy (325 mg), ARAMIS employed dual antiplatelet therapy with clopidogrel (300 mg loading, then 75 mg/day) plus aspirin (100 mg/day) for 12 days, while TEMPO-2 and PUMICE allowed investigator to determine antiplatelet regimens, with the majority of patients in PUMICE receiving aspirin plus clopidogrel (90% of standard care patients). The adoption of DAPT, as validated in the CHANCE trial, in the control arms of more recent trials may have improved outcomes in the comparator group, raising the threshold for IVT to demonstrate superiority (28). Despite these pharmacological differences, the consistently low heterogeneity across all pooled outcomes and the stability of our leave-one-out sensitivity analyses suggest that these variations did not compromise the robustness of our findings.

Another important consideration is the variation in stroke etiology according to the TOAST classification across the included trials. As shown in Table 1, only the ARAMIS and PUMICE reported TOAST subtype data (12, 14), while PRISMS and TEMPO-2 did not (11, 13). In the ARAMIS trial, the majority of patients had undetermined etiology (approximately 62%), with small artery occlusion accounting for approximately 23% (12). In contrast, in the PUMICE trial, small artery occlusion predominated (approximately 54%), followed by large artery atherosclerosis (approximately 40%), while cardioembolic stroke was notably rare (<3%) (14). These differences in stroke subtype distribution may influence the response to thrombolytic therapy. Stroke etiology is a recognized predictor of post-thrombolysis outcomes; in particular, cardioembolic stroke has been associated with a higher risk of hemorrhagic transformation. Ahmed et al. (29) demonstrated that in atrial fibrillation patients with embolic stroke treated with alteplase, older age, higher NIHSS, sustained atrial fibrillation, warfarin use, and higher HAS-BLED score were independent predictors of different ECASS-based subtypes of hemorrhagic transformation. Furthermore, Zeinhom et al. (30) reported that post-thrombolysis intracranial hemorrhage in atrial fibrillation patients carries lasting prognostic consequences, with unfavorable functional outcomes persisting at 2-year follow-up. Given the low proportion of cardioembolic strokes in our pooled sample, the generalizability of our findings to patients with cardioembolic minor stroke remains uncertain and warrants further investigation. Additionally, the inability to perform subgroup analyses by TOAST classification due to incomplete reporting represents a limitation that future trials should address.

Overall, the results of all trials showed that intravenous thrombolytic therapy did not benefit the patients, and in TEMPO-2 it was found that the number of deaths was higher in the tenecteplase group (5%) than in the control group (1%), *P* < 0.01. Although in several recent Meta-analyses, some researchers had also compared alteplase with antiplatelet therapy, and their findings suggested that IVT had not been shown to be beneficial (31–33). However, it was worth noting that observational studies were included in each meta-analysis, making it much more heterogeneous with a much higher risk of bias. Because of this, the findings were not convincing. Similarly, the study by Qin et al. (34). included a total of 3,975 patients, comprising two randomized controlled trials and four observational studies, and was subject to the same potential risk of confounding bias. Significant heterogeneity was observed in the primary 90-day functional outcomes (I^2^ = 65%). Their findings supported the association between early antiplatelet therapy and a reduced risk of sICH (34). In the 2019 update, minor stroke was explicitly addressed as a separate category, and three new recommendations were introduced. For patients with minor but disabling ischemic stroke symptoms who are eligible for treatment within 3–4.5 h of symptom onset—or have a known time of last being well—intravenous thrombolysis may be considered reasonable. However, it is important to note that for patients with mild non-disabling stroke symptoms (NIHSS score 0–5), intravenous alteplase is not recommended, whether they present within 3 h or between 3 and 4.5 h after symptom onset, or have a clearly documented last-known-well time (7, 8). Therefore, the assessment of disabling deficits in minor ischemic stroke is critically important. Such assessment should include, but is not limited to: complete hemianopia, severe aphasia, visual neglect, significant motor weakness (e.g., inability to sustain effort against gravity), symptoms that may interfere with basic activities of daily living or return to work, and any condition considered potentially disabling by the patient, family, or treating physician (35).

Therefore, while waiting for more sufficient evidence to provide clearer clinical guidance, we believe that intravenous thrombolysis in patients with minor stroke should be balanced with multiple factors, and that antiplatelet therapy is more beneficial and safer. Levine et al. (36) suggested that the disabling nature of the symptoms and the time of onset of symptoms should be taken into account (the earlier the use of alteplase, the greater the expected benefit). But again, other factors included the increased risk of bleeding with IV thrombolysis, prior history of antiplatelet therapy, other baseline functional status, and so on (25). It is worth noting, however, that despite the increased risk of bleeding in the brain with IV thrombolysis, the overall incidence of symptomatic intracranial hemorrhage is very low (25).

## Limitations

This study still has some limitations. First, although the trial group was all intravenous thrombolysis, three pharmacologically distinct thrombolytic agents were included (alteplase, tenecteplase, and prourokinase), which differ in fibrin specificity, half-life, and dosing regimen; however, the consistently low heterogeneity (I^2^ = 0%) across all outcomes provides reassurance regarding the robustness of the pooled estimates. Second, the standard treatment protocols for antiplatelet therapy were not uniform, ranging from aspirin monotherapy to dual antiplatelet therapy and investigator-determined regimens, which may have differentially influenced the control-arm outcomes. Third, not all included trials reported TOAST classification data, precluding subgroup analysis by stroke etiology. Third, due to the lack of stratified aggregate data in some included trials (such as TEMPO-2 and PUMICE), we were unable to perform specific subgroup analyses comparing disabling versus non-disabling mild stroke patients, which limits the granularity of our findings regarding symptom severity. Fourth, future studies are needed to specifically address combined treatment strategies, such as acute ischemic stroke cases involving endovascular therapy, in order to draw more valuable and comprehensive conclusions. We still look forward to more clinical randomized controlled trials to validate our findings in the future.

## Conclusion

The results of this systematic review and meta-analysis suggest that intravenous thrombolysis decreases the incidence of functional independence and elevates 90-day mortality as well as the incidence of sICH in patients with mild AIS compared with antiplatelet standard therapy. Therefore, we recommend antiplatelet standard therapy rather than intravenous thrombolysis for patients with mild AIS.

## Data Availability

The original contributions presented in this study are included in this article/Supplementary material, further inquiries can be directed to the corresponding authors.
