# Correction: Intravenous thrombolysis versus antiplatelet standard care for patients with mild acute ischemic stroke: a systematic review and meta-analysis

**DOI:** 10.3389/fmed.2026.1828213

**Published:** 2026-03-27

**Authors:** Yitao Zhou, Yangbin Zhou, Ganying Hunag, Hongmei Wang

**Affiliations:** 1 School of Nursing, Zhejiang Chinese Medical University, Hangzhou, Zhejiang, China; 2 Department of Emergency, Affiliated Hangzhou First People's Hospital, School of Medicine, Westlake University, Hangzhou, Zhejiang, China

**Keywords:** antiplatelet standard care, intravenous thrombolysis, meta-analysis, mild acute ischemic stroke, review

Author Yitao Zhou was erroneously assigned to affiliation Department of Emergency, Affiliated Hangzhou First People's Hospital, School of Medicine, Westlake University, Hangzhou, Zhejiang, China. This affiliation has now been removed for author Yitao Zhou, and the correct affiliation School of Nursing, Zhejiang Chinese Medical University, Hangzhou, Zhejiang, China has been assigned to the author.

The original version of this article has been updated.

