# Supplementary material for: Intravenous thrombolysis versus antiplatelet standard care for patients with mild acute ischemic stroke: a systematic review and meta-analysis
Source: Front Med (Lausanne). 2026 Mar 11;13:1780490. doi: 10.3389/fmed.2026.1780490 (PMC13013522; doi:10.3389/fmed.2026.1780490)
Supplement: Supplementary file 1 [file Data_Sheet_1.docx]

**Supplementary 1 Online Content**

**eTable1. The complete search strategy.**

**eFigure 1. Quality Assessment of RCTs**

**eFigure 2. Influence Analysis in Meta-Analysis Using Leave-One-Out Method (mRS ≤ 1)**

**eFigure 3. Influence Analysis in Meta-Analysis Using Leave-One-Out Method (mRS ≤ 2)**

**eFigure 4. Influence Analysis in Meta-Analysis Using Leave-One-Out Method (90-day mortality)**

**eFigure 5. Influence Analysis in Meta-Analysis Using Leave-One-Out Method (sICH)**

**eTable1. The complete search strategy.**

| **Database** | **Detailed Search Strategy** | **Date** | **Results** |
| --- | --- | --- | --- |
| Pubmed | "ischemic stroke"[MeSH Terms] OR ("ischemic"[All Fields] AND "stroke"[All Fields]) OR "ischemic stroke"[All Fields]) AND (("intraveneous"[All Fields] OR "intraveneously"[All Fields] OR "intravenous"[All Fields] OR "intravenously"[All Fields]) AND "thrombolysis"[All Fields]) AND ("antiplatelet"[All Fields] OR "antiplatelets"[All Fields] | June 27, 2025 | 231 |
| Cochrane Library | ischemic stroke:ti,ab,kw AND intravenous thrombolysis:ti,ab,kw AND antiplatelet:ti,ab,kw (Word variations have been searched) | June 27, 2025 | 75 |
| Embase | ('ischemic stroke'/exp OR 'ischemic stroke' OR (ischemic AND ('stroke'/exp OR stroke))) AND intravenous AND thrombolysis AND antiplatelet | June 27, 2025 | 557 |
| Web of Science | ischemic stroke (Topic) and intravenous thrombolysis (Topic) and antiplatelet (Topic)  https://www.webofscience.com/wos/alldb/summary/362be144-b6fa-46da-a8db-58a0c1112dad-01463c51c1/relevance/1 | June 27, 2025 | 413 |


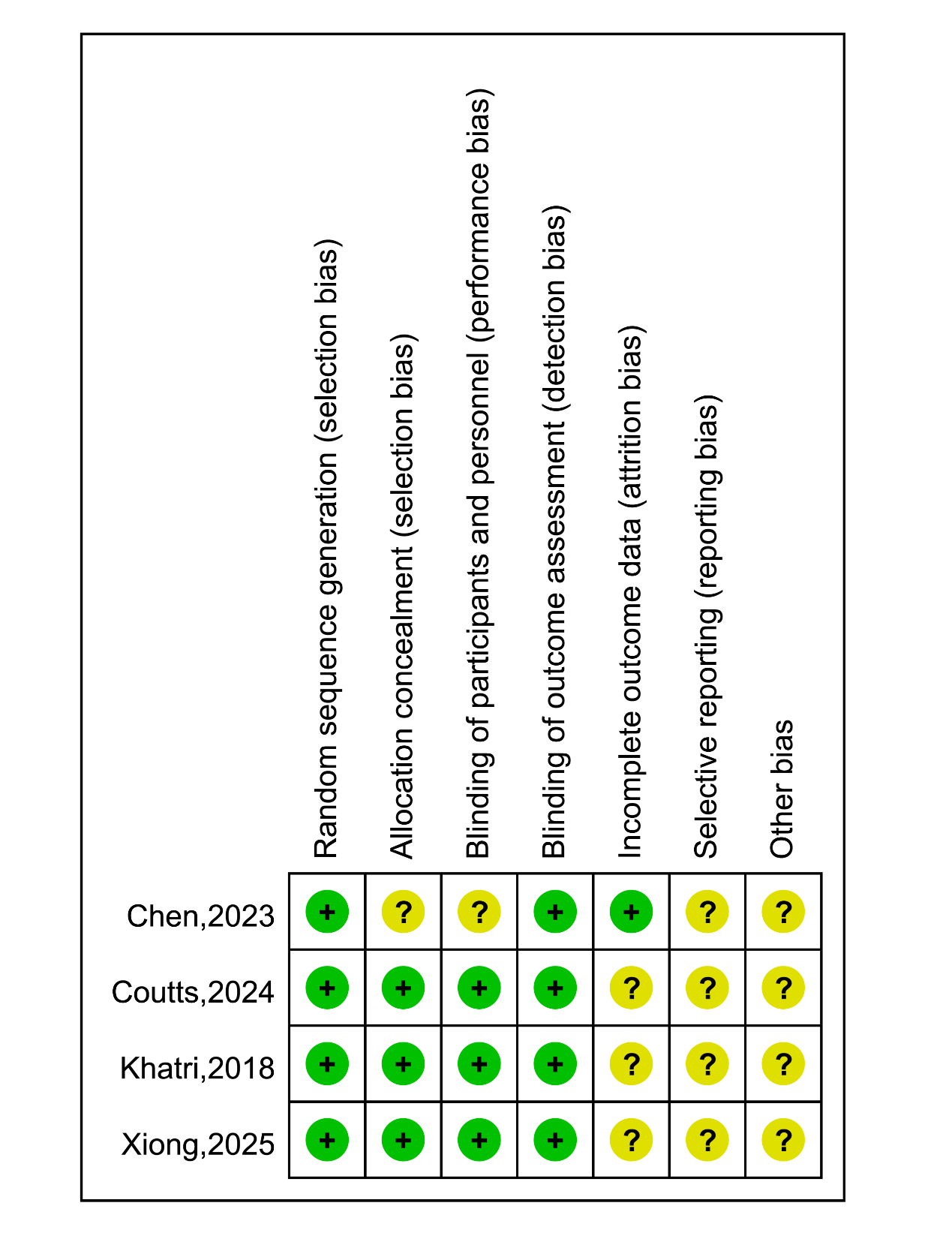


**eFigure 1. Quality Assessment of RCTs**


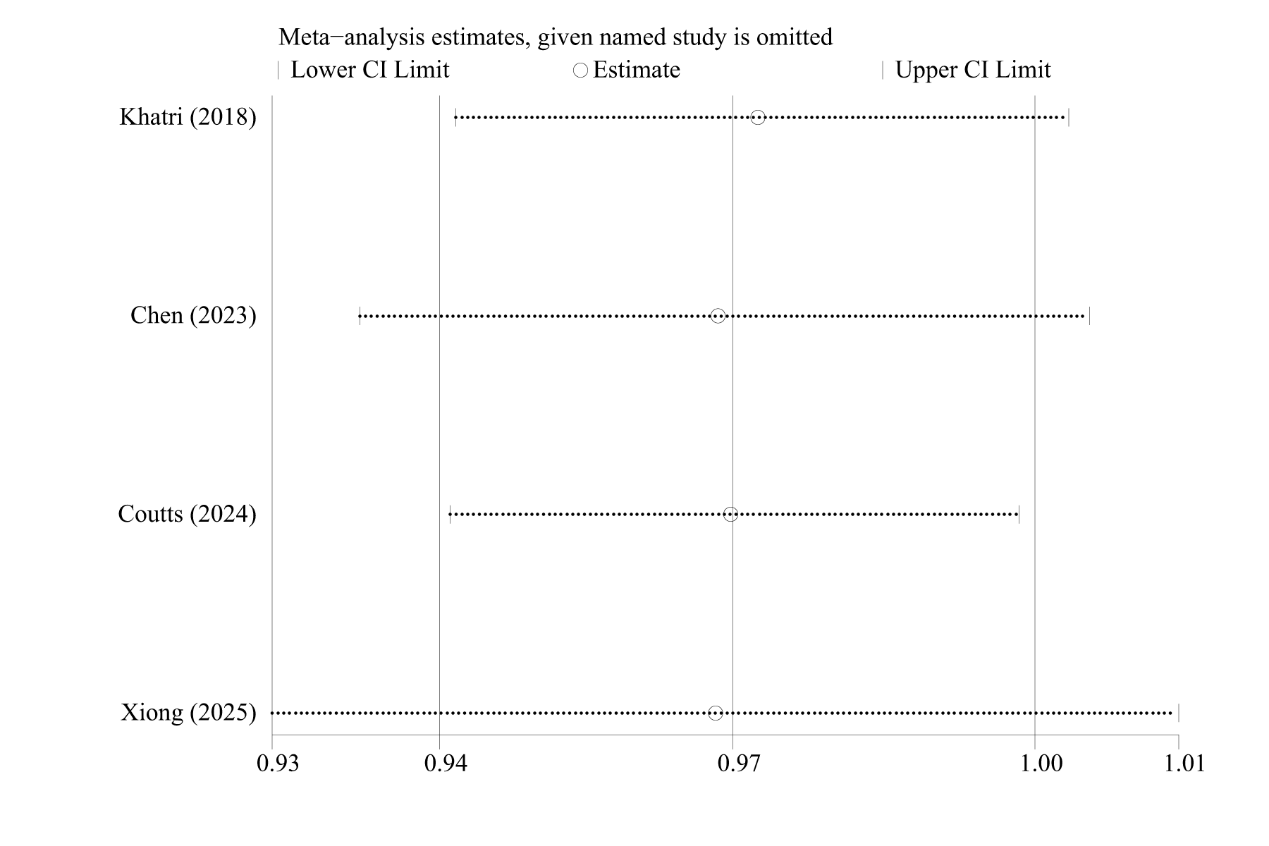


**eFigure 2. Influence Analysis in Meta-Analysis Using Leave-One-Out Method (mRS ≤ 1)**


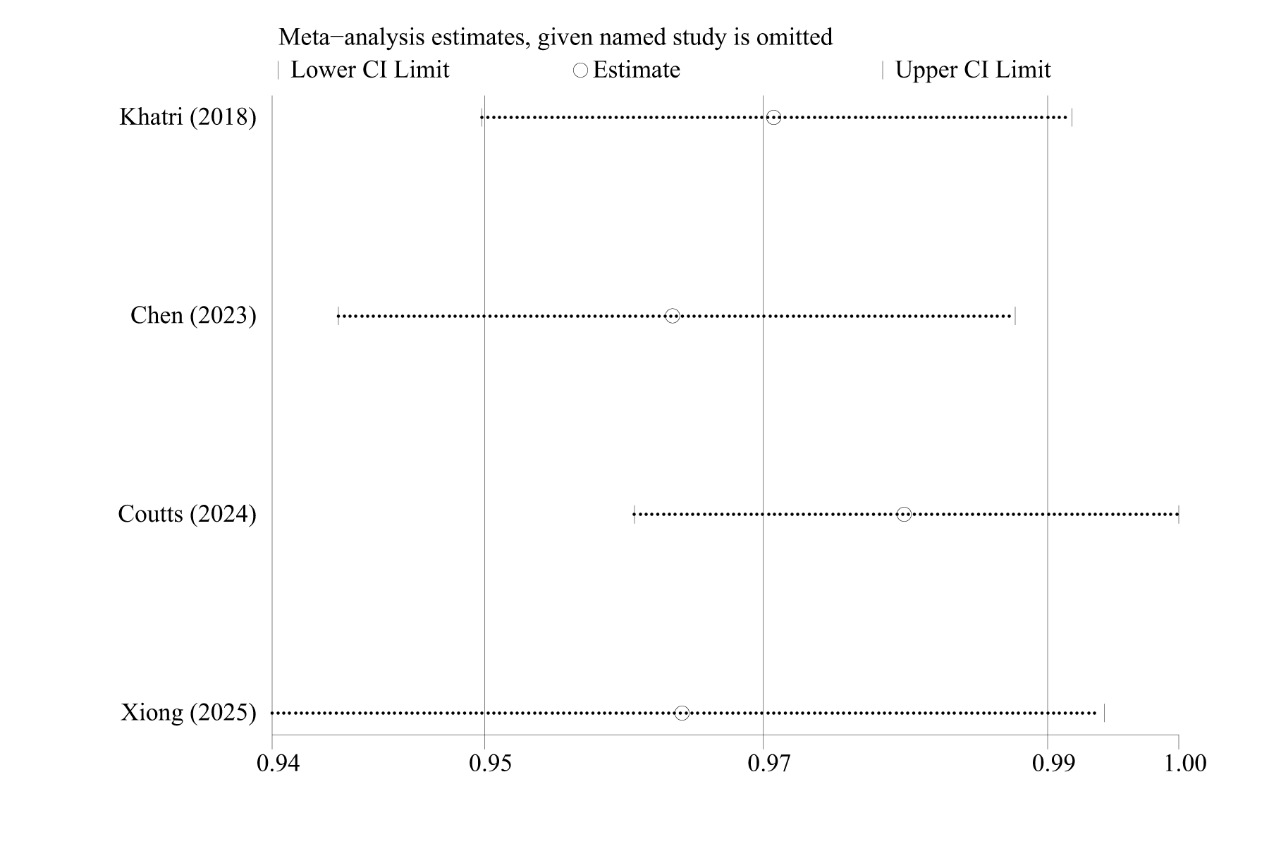


**eFigure 3. Influence Analysis in Meta-Analysis Using Leave-One-Out Method (mRS ≤ 2)**


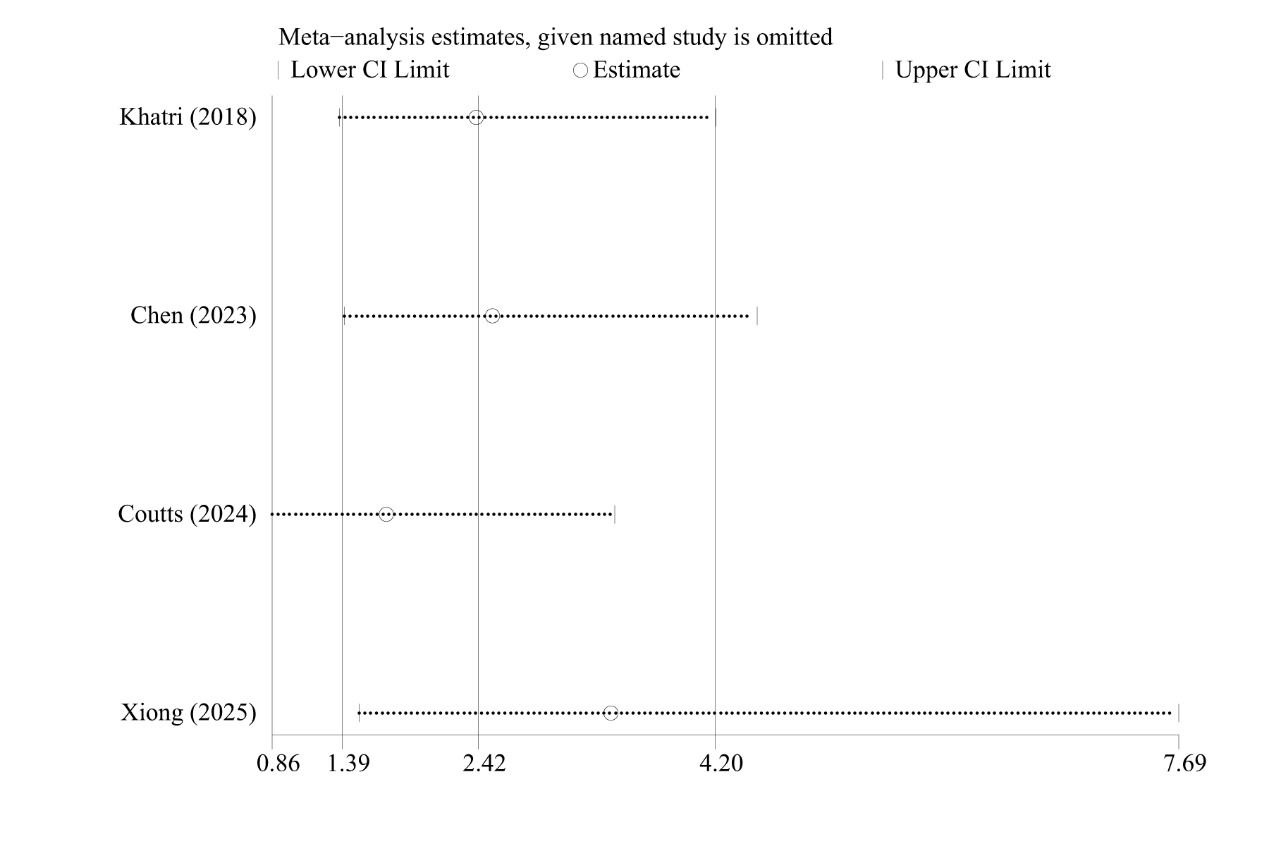


**eFigure 4. Influence Analysis in Meta-Analysis Using Leave-One-Out Method (90-day mortality)**


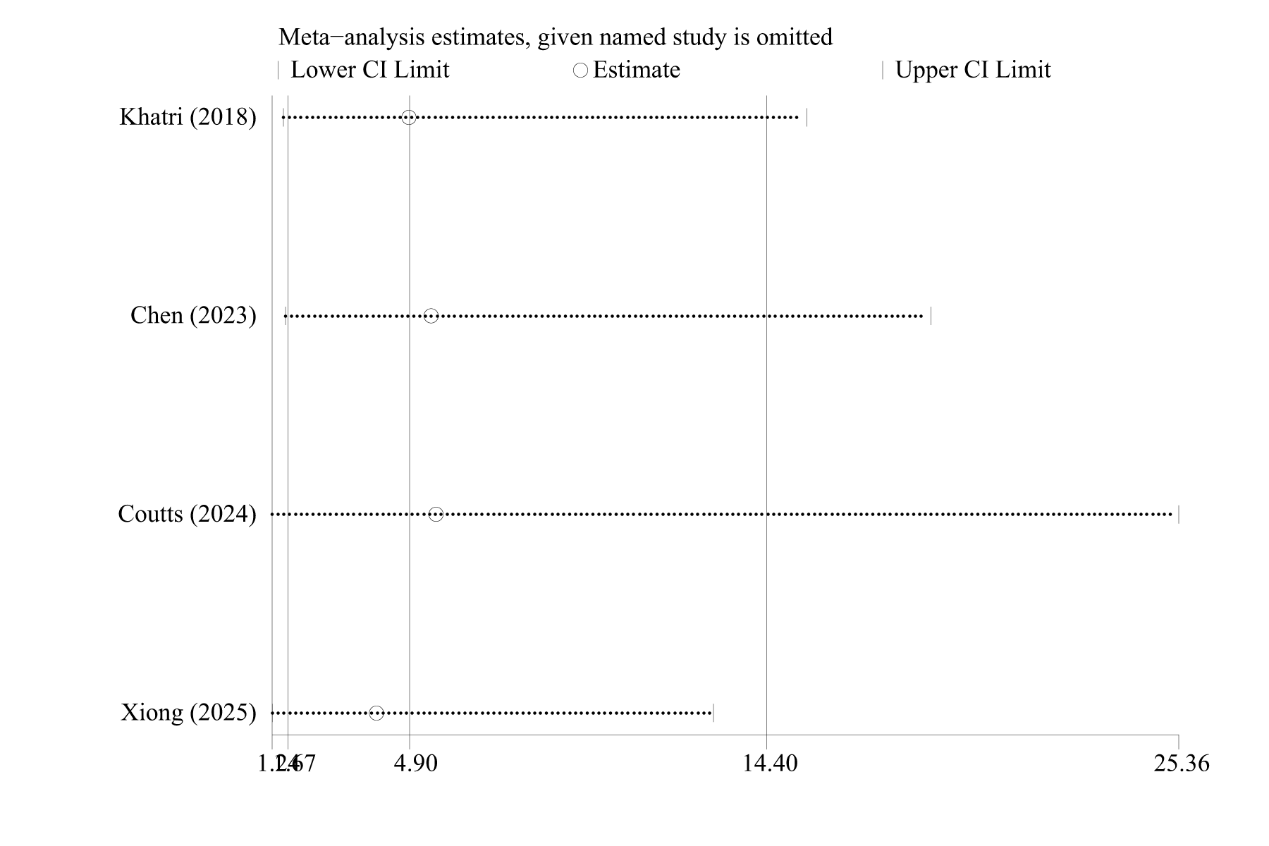


**eFigure 5. Influence Analysis in Meta-Analysis Using Leave-One-Out Method (sICH)**
